# Interfacial Energy Level Tuning for Efficient and Thermostable CsPbI_2_Br Perovskite Solar Cells

**DOI:** 10.1002/advs.201901952

**Published:** 2019-09-30

**Authors:** En‐Chi Shen, Jing‐De Chen, Yu Tian, Yu‐Xin Luo, Yang Shen, Qi Sun, Teng‐Yu Jin, Guo‐Zheng Shi, Yan‐Qing Li, Jian‐Xin Tang

**Affiliations:** ^1^ Jiangsu Key Laboratory for Carbon‐Based Functional Materials & Devices Institute of Functional Nano & Soft Materials (FUNSOM) Soochow University 199 Ren'ai Road Suzhou 215123 Jiangsu China; ^2^ School of Physics and Electronics Science Ministry of Education Nanophotonics & Advanced Instrument Engineering Research Center East China Normal University Shanghai 200062 China; ^3^ Institute of Organic Optoelectronics (IOO) JITRI Wujiang Suzhou 215215 China

**Keywords:** all‐inorganic perovskite solar cells, energy level alignment, flexible perovskite solar cells, thermal stability

## Abstract

Inorganic mixed‐halide CsPbX_3_‐based perovskite solar cells (PeSCs) are emerging as one of the most promising types of PeSCs on account of their thermostability compared to organic–inorganic hybrid counterparts. However, dissatisfactory device performance and high processing temperature impede their development for viable applications. Herein, a facile route is presented for tuning the energy levels and electrical properties of sol–gel‐derived ZnO electron transport material (ETM) via the doping of a classical alkali metal carbonate Cs_2_CO_3_. Compared to bare ZnO, Cs_2_CO_3_‐doped ZnO possesses more favorable interface energetics in contact with the CsPbI_2_Br perovskite layer, which can reduce the ohmic loss to a negligible level. The optimized PeSCs achieve an improved open‐circuit voltage of 1.28 V, together with an increase in fill factor and short‐circuit current. The optimized power conversion efficiencies of 16.42% and 14.82% are realized on rigid glass substrate and flexible plastic substrate, respectively. A high thermostability can be simultaneously obtained via defect passivation at the Cs_2_CO_3_‐doped ZnO/CsPbI_2_Br interface, and 81% of the initial efficiency is retained after aging for 200 h at 85 °C.

## Graphical abstract

An all‐inorganic mixed‐halide perovskite solar cell with a power conversion efficiency of 16.42% is realized by using a Cs_2_CO_3_‐doped ZnO electron transport layer, which ascribes to the interfacial energy level tuning for reducing ohmic loss at the contact and enlarging the built‐in potential. A high thermostability is simultaneously obtained via surface defect passivation for improving the CsPbI_2_Br film against phase transformation.

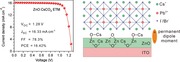

## 1 Introduction

Metal halide perovskite solar cells (PeSCs) have attracted tremendous attention as one of the most promising candidates for next‐generation photovoltaic technologies in the past decade.^1^, ^2^, ^3^, ^4^, ^5^, ^6^, ^7^, ^8^, ^9^, ^10^ The power conversion efficiency (PCE) of PeSCs has already broken through 24% in single‐junction architectures,^10^ and up to 28% in silicon‐based tandem cells. Despite the rapid progress in efficiency, the practical application of PeSCs is impeded by intrinsic volatility and thermal instability of organic–inorganic hybrid perovskite materials. Partial or complete substitution of organic cations (e.g., (NH_2_)_2_CH^+^ or CH_3_NH_3_^+^) is becoming an effective strategy to realize the stable PeSCs. Especially, all‐inorganic perovskites with Cs^+^ as A‐site cation (i.e., CsPbX_3_, X = Cl, Br, and I) hold a great potential to replace the organic–inorganic hybrid counterpart for photovoltaic applications, since CsPbI_3_ and CsPbBr_3_ perovskites can be compositionally stable even over 300 °C.^11^, ^12^, ^13^, ^14^

Among various CsPbX_3_ perovskites, CsPbI_3_ in cubic crystal structure benefits the sufficient absorption of visible light due to the smallest optical bandgap (*E*_g_) of ≈1.73 eV (α‐phase). Unfortunately, CsPbI_3_ PeSCs are suffering from the device instability due to severe octahedral rotation distortion and spontaneous transformation from α‐phase to an undesired δ‐phase at room temperature.^15^, ^16^, ^17^ Notably, CsPbBr_3_ with small Br^−^ ions shows the superior phase stability, while its wide *E*_g_ of ≈2.3 eV restricts the light‐harvesting capacity in the visible light region.^18^, ^19^, ^20^, ^21^ In this regard, the compositional optimization of all‐inorganic CsPbX_3_ perovskites indicates that mixed‐halide CsPbI_2_Br with a suitable *E*_g_ of ≈1.92 eV may balance the light‐harvesting efficiency and structural stability under ambient conditions for achieving highly efficient and stable PeSCs.^11^, ^21^, ^22^, ^23^, ^24^ Upon many efforts dedicated to morphology control and surface passivation,^21^, ^22^, ^23^, ^25^ the performance of CsPbI_2_Br PeSCs has been impressively improved with an encouraging PCE of 16.2% and good photostability.^26^

Nevertheless, the PCE of CsPbI_2_Br PeSCs is still uncompetitive to that of the typical hybrid perovskite counterparts, and it is highly desirable to develop new methods to further optimize the perovskite layers and device structures. To date, interface energetics in CsPbI_2_Br PeSCs becomes one bottleneck of the PCE enhancement.^27^, ^28^ The large energy level offsets between CsPbI_2_Br perovskite and charge transport layers induce the charge accumulation at the interfaces, leading to the undesirable recombination loss.^29^ Consequently, unsatisfactory device performance is observed in CsPbI_2_Br PeSCs, especially with an open‐circuit voltage (*V*_OC_) lower than 1.2 V. Compared to the *E*_g_ value of CsPbI_2_Br (≈1.92 eV), severe energy loss over 0.72 eV occurs in commonly reported CsPbI_2_Br PeSCs.^11^, ^21^, ^22^, ^23^, ^24^, ^25^ A wide variety of solutions have recently been reported to tune the interfacial energy levels to overcome this adverse impact.^24^, ^26^, ^27^, ^28^, ^29^, ^30^ The majority of these methods focused on the partial substitution of the components in CsPbI_2_Br, in which a small quantity of F, Sn, or Ge was introduced to match the conduction band alignment between perovskite and electron transport material (ETM).^24^, ^28^, ^30^ However, engineering the CsPbI_2_Br perovskites may cause the unexpected change in the film quality and optical properties. Until now, only a few pertinent studies have been conducted to tune the energy levels of charge transport layers,^26^ which seems to be a universal and facile method to reduce the interfacial energy level offset.

Herein, for the first time, we employ the sol–gel‐derived ZnO as an ETM for all‐inorganic CsPbI_2_Br PeSCs with a device architecture of indium‐tin‐oxide (ITO)/ZnO ETM/CsPbI_2_Br/2,29,7,70‐tetrakis‐(*N*,*N*‐di‐*p*‐methoxyphenyl‐amine)‐9,99‐spirobifluorene (Spiro‐OMeTAD)/MoO_3_/Ag. It is noted that ZnO has better electrical and optical properties than the commonly used TiO_2_.^31^, ^32^, ^33^, ^34^ Moreover, the low processing temperature of sol–gel‐derived ZnO makes it a universal material for different applications, in particular, which have temperature limitation. Above all, the energy level of ZnO can be tuned easily by introducing organic or inorganic materials as a dopant,^35^, ^36^ which benefits the addressing of energy level offset issue in PeSCs. In particular, the use of alkali metal compounds is an effective n‐type doping method due to the advantages of the enhanced electrical properties, ease of material handling, and operational stability.^37^ In this work, the energy levels and electrical properties of sol–gel‐derived ZnO ETM are tuned via the doping of classical alkali metal compounds, including cesium carbonate (Cs_2_CO_3_), cesium fluoride (CsF), and potassium carbonate (K_2_CO_3_). Experimental findings demonstrate that Cs_2_CO_3_ shows the best doping effect on ZnO ETMs among three dopants. The doping of Cs_2_CO_3_ with proper quantity can effectively tune the energetics of ZnO ETM and improve the electron transport property. Particularly, Cs_2_CO_3_‐doped ZnO (denoted as ZnO:Cs_2_CO_3_) induces an energy level offset of 0.1 eV relative to the conduction band of CsPbI_2_Br perovskite, reducing the ohmic loss to a negligible level. The *V*_OC_ is significantly boosted to 1.28 V along with a simultaneous increase in fill factor (FF) and short‐circuit current density (*J*_SC_). As a result, the optimized PCE of 16.42% is achieved for CsPbI_2_Br PeSCs, which is one of the highest efficiencies reported for all‐inorganic PeSCs to date. In addition, the low‐temperature processing of ZnO ETM enables the realization of flexible CsPbI_2_Br PeSCs on plastic substrate, showing a record PCE of 14.82%. The flexible PeSC can remain 81% of its initial PCE after 1000 cycles of mechanical bending. Moreover, the ZnO:Cs_2_CO_3_ ETM provides a stabilization effect on CsPbI_2_Br PeSCs against phase transformation. Encouragingly, a high thermostability can be simultaneously obtained via defect passivation at the ZnO:Cs_2_CO_3_/CsPbI_2_Br interface, and the optimized devices retain 96% and 81% of their initial efficiencies after aging for 200 h at room temperature and 85 °C, respectively.

## 2 Results and Discussion

**Figure 1:**
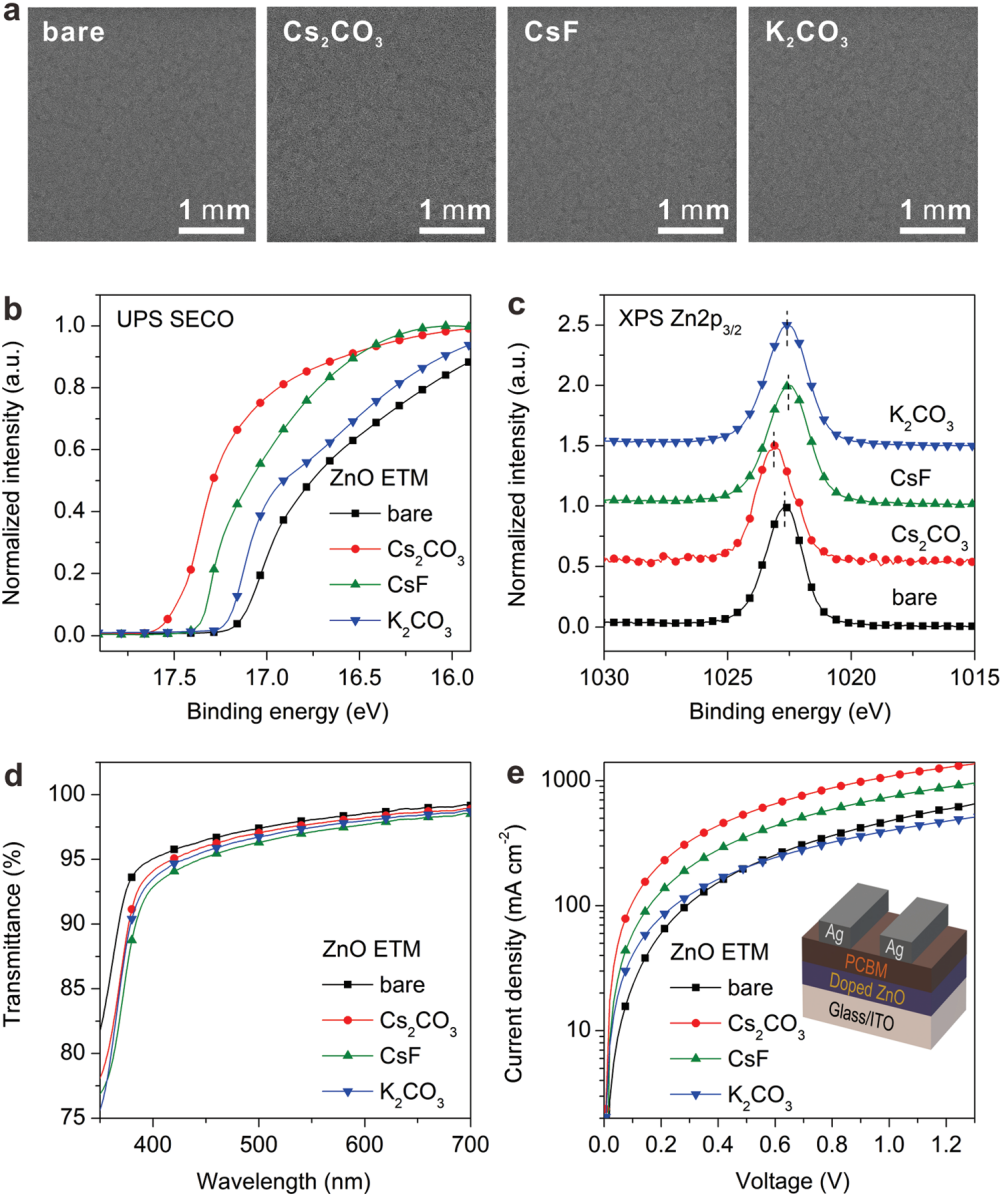
Characterization of doped ZnO ETMs. a) SEM images, b) UPS spectra in the SECO region, c) XPS Zn2P_3/2_ core level spectra, d) optical transmission spectra, and e) *J*–*V* curves of electron‐only devices.

For comparison, the doping was performed by adding the same concentration (4 mol%) of Cs_2_CO_3_, CsF, and K_2_CO_3_ into the ZnO precursors, while the preparation procedure of sol–gel‐derived ZnO films was kept the identical (see the details in the “Experimental Section”). The doping effect of various dopants on ZnO ETMs was investigated by morphological, electronic, and optical measurements. **Figure** **1**a displays the scanning electron microscopy (SEM) images of bare and doped ZnO films. It is noted that there is no significant difference in surface morphology among these samples, and the uniform structures rule out the doping influence on the growth of ZnO films. The electronic structures of various ZnO ETMs were characterized by ultraviolet and X‐ray photoemission spectroscopies (UPS and XPS). Figure S1 (Supporting Information) displays the XPS survey spectra, confirming the presence of various dopants in the sol–gel‐derived ZnO films. Moreover, the energy‐dispersive X‐ray spectroscopy (EDX) images of ZnO:Cs_2_CO_3_ ETM (Figure S2, Supporting Information) show a relatively uniform distribution of each element, manifesting the doping uniformity in ZnO films. Figure 1b shows the secondary electron cutoff (SECO) region of the UPS spectra, from which the corresponding work functions of ZnO films (φ_ZnO_) can be estimated to be 4.0, 3.7, 3.85, and 4.05 eV, respectively, for bare ZnO, ZnO:Cs_2_CO_3_, CsF‐doped ZnO (ZnO:CsF), and K_2_CO_3_‐doped ZnO (ZnO:K_2_CO_3_). A decrease in φ_ZnO_ implies the reduced energy level offset between the conduction bands of ZnO and CsPbI_2_Br. Particularly, the Cs_2_CO_3_ doping will cause a better energy level alignment than that of CsF and K_2_CO_3_. The XPS spectra of Zn2P_3/2_ core level (Figure 1c) indicate no observable variation in the spectral shape, but a clear peak shift for doped ZnO films. Moreover, the shift trend among various ZnO films is coincident with the variation of φ_ZnO_. It is thus reasonable to infer that no chemical reaction occurs between dopants and ZnO except for the change of φ_ZnO_. Figure 1d shows the optical transmittance spectra of various ZnO films. It is observed that the optical transmittance of doped ZnO films is slightly lower than that of bare ZnO, which might be due to the light absorption of different dopants. However, all the ZnO films have a transmittance higher than 95% at 500 nm.

To verify the electron transport capability of various ZnO films, electron‐only devices with a device structure of glass/ITO/ZnO/[6,6]‐phenyl C61 butyric acid methyl ester (PCBM)/Ag were fabricated, in which PCBM was used to facilitate the electron injection from Ag electrode into ZnO. The recorded current density–voltage (*J*–*V*) curves of various devices are plotted in Figure 1e. A higher current density is observed for the doping of Cs_2_CO_3_ and CsF in ZnO films. Especially, the current density of ZnO:Cs_2_CO_3_‐based device is approaching one order of magnitude higher than that of bare ZnO. It is thus concluded that the doping Cs_2_CO_3_ and CsF in ZnO can dramatically improve the electron transport capability, which will benefit the electron transfer and extraction in PeSCs. As a comparison, there is no clear change on *J*–*V* characteristics when incorporating K_2_CO_3_ into ZnO. The corresponding current density under high bias voltage is even lower than that of bare ZnO, indicating that the doping of K_2_CO_3_ gives rise to an increase in trap states and hinders the electron transport.^38^ In addition, the contact angles of bare and doped ZnO ETMs were measured to explore the doping effect on the surface wetting property. As illustrated in Figure S3 (Supporting Information), the doping of Cs_2_CO_3_ reduces the contact angle by ≈7°, while both K_2_CO_3_ and CsF lead to an increase in contact angle. Particularly, a large contact angle of 45.9° is observed for ZnO:K_2_CO_3_. These findings indicate that the ZnO:Cs_2_CO_3_ ETM may enable a better physical contact with CsPbI_2_Br perovskite for better film growth and electron extraction.^39^

**Figure 2:**
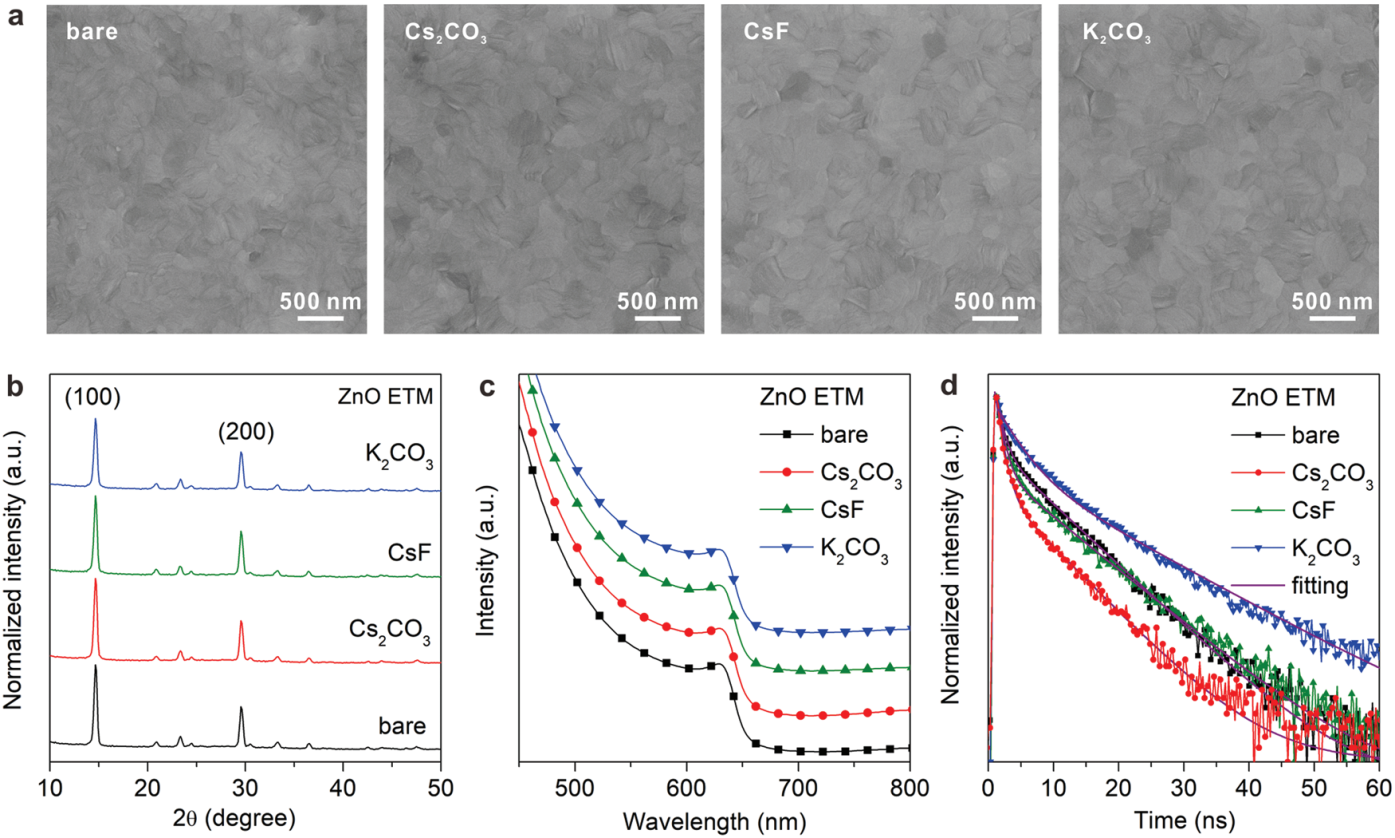
a) SEM images, b) XRD patterns, c) absorption spectra, and d) PL decay curves of CsPbI_2_Br perovskite films formed on different ZnO substrates.

To gain insight into the doping effects for the perovskite films and related devices, the properties of CsPbI_2_Br perovskite layers deposited on various ZnO ETMs were characterized, and a two‐step method with low‐temperature annealing procedure was used to fabricate the CsPbI_2_Br films. **Figure** **2**a presents the SEM images of CsPbI_2_Br films deposited on different ZnO ETMs. Perovskite films with uniform and pinhole‐free morphology were observed in all cases, indicating that the film formation was barely affected by the doping. Further structural inspection of the CsPbI_2_Br films was conducted by X‐ray diffraction (XRD) measurements. As shown in Figure 2b, all the samples display two feature peaks at 2θ = 14.4° and 29.3°, which correspond to the (100) and (200) planes of CsPbI_2_Br in cubic crystal structure.^25^ The identical diffraction patterns for perovskite films deposited on different ZnO substrates suggest that the crystalline structure of CsPbI_2_Br is not affected by the different dopants. Moreover, the absorption spectra of perovskite films on different ZnO ETMs (Figure 2c) are almost identical to each other, exhibiting the same absorption onsets. Intriguingly, the time‐resolved photoluminescence (TRPL) decay curves for CsPbI_2_Br on various ZnO ETMs exhibit different behaviors (Figure 2d). The TRPL lifetimes of CsPbI_2_Br films on various substrates were calculated with a biexponential fitting to the TRPL spectra, showing the lifetimes of 6.6, 4.2, 6.3, and 10.2 ns for bare ZnO, ZnO:Cs_2_CO_3_, ZnO:CsF, and ZnO:K_2_CO_3_, respectively. It is evident that the fastest PL decay is obtained for CsPbI_2_Br on ZnO:Cs_2_CO_3_, implying that electrons can be more efficiently extracted from CsPbI_2_Br to ZnO:Cs_2_CO_3_ than the others.^7^ In contrast, the electron extraction from the perovskite layer to ZnO:K_2_CO_3_ is severely retarded due to low electron conductivity as indicated in Figure 1e.

**Figure 3:**
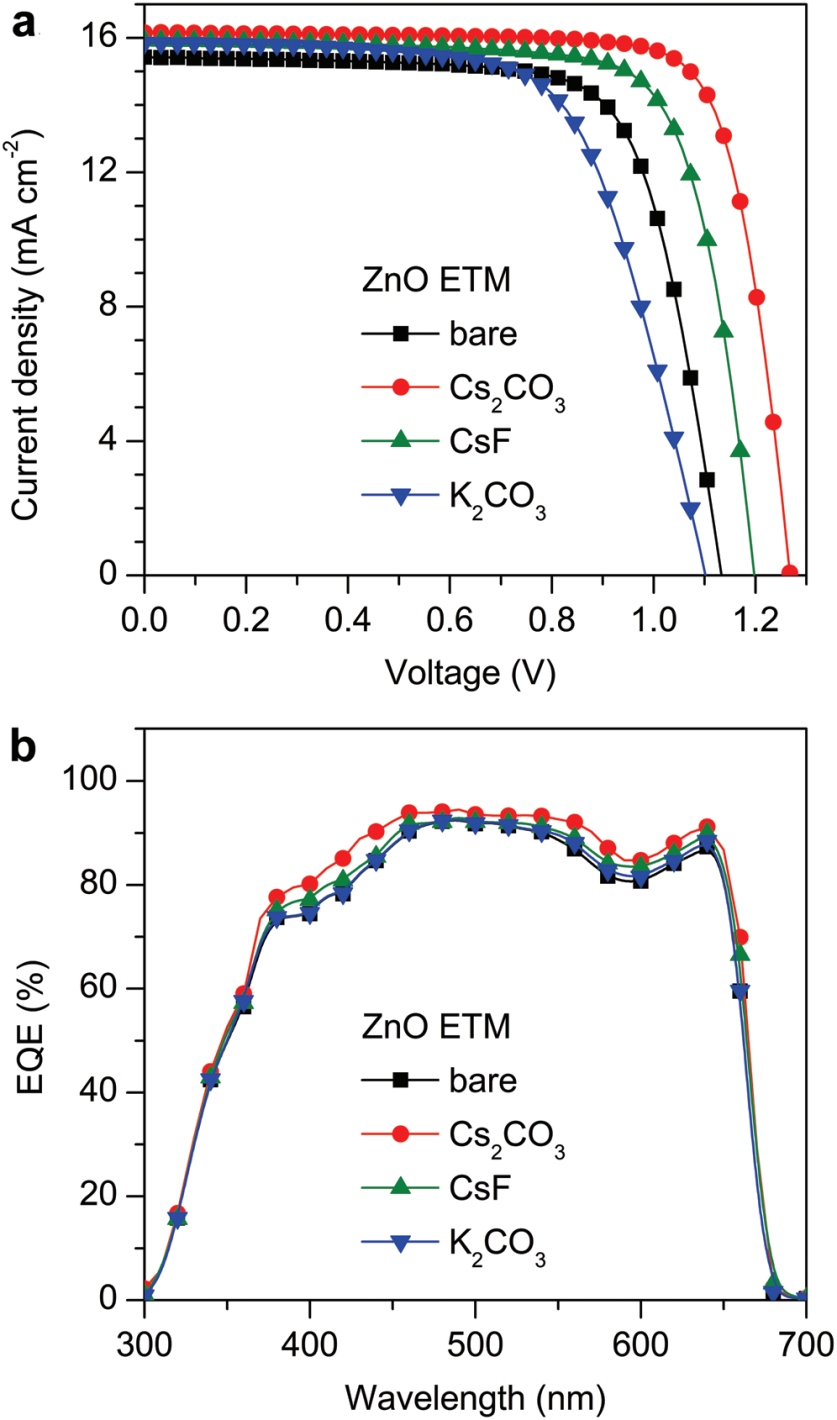
a) *J*–*V* curves and b) EQE spectra of CsPbI_2_Br PeSCs with different ZnO ETMs under 100 mW cm^−2^ AM 1.5G illumination.

To examine the effect of doped ZnO ETMs on device performance, all‐inorganic PeSCs were fabricated with a planar n–i–p structure of ITO/ZnO ETM/CsPbI_2_Br/Spiro‐OMeTAD/MoO_3_/Ag. The doping concentrations of Cs_2_CO_3_, CsF, and K_2_CO_3_ were varied from 0 to 6 mol% for the performance optimization (Figure S4, Supporting Information), yielding an optimal concentration of 4 mol%. The *J*–*V* characteristics of the CsPbI_2_Br PeSCs based on the optimized ZnO ETMs are depicted in **Figure** **3**a and **Table** **1**. The reference device based on bare ZnO shows the *V*_OC_ of 1.13 V, *J*_SC_ of 15.42 mA cm^−2^, FF of 72.2%, and PCE of 12.68%. As a comparison, the CsF doping causes a modulate enhancement of 14.34% in PCE, whereas the PCE is declined to 11.48% with the use of K_2_CO_3_. Notably, the use of ZnO:Cs_2_CO_3_ results in the maximal PCE of 16.42%, which is one of the highest efficiencies reported in the literatures for inorganic PeSCs.^26^ Moreover, the ZnO:Cs_2_CO_3_‐based CsPbI_2_Br PeSCs exhibit a high reproducibility (Figure S5, Supporting Information) and slight hysteresis (Figure S6, Supporting Information). As summarized in Table 1, the average PCEs are 12.14%, 16.10%, 13.71%, and 10.86%, respectively, for CsPbI_2_Br PeSCs based on bare ZnO, ZnO:Cs_2_CO_3_, ZnO:CsF, and ZnO:K_2_CO_3_. It is confirmed that the ZnO:Cs_2_CO_3_‐based devices exhibit the best performance. The external quantum efficiency (EQE) spectra were recorded to verify the variation in *J*_SC_ for different CsPbI_2_Br PeSCs (Figure 3b). The integration of the EQE spectra and solar photon flux yields the *J*_SC_ values of 14.85, 15.64, 15.24, and 14.93 mA cm^−2^ for CsPbI_2_Br PeSCs based on bare ZnO, ZnO:Cs_2_CO_3_, ZnO:CsF, and ZnO:K_2_CO_3_, respectively. The calculated *J*_SC_ values are in agreement with the experimentally measured results (Figure 3a) with an error below 5%. Taking into account the TRPL decay profiles (Figure 2d), the variation in EQE can be ascribed to the difference in electron extraction capability for various ZnO ETMs. The stabilized power outputs of various PeSCs were also recorded and shown in Figure S7 (Supporting Information). The results are nearly identical to the parameters extracted from the *J*–*V* curves, which further confirm the positive influence of Cs_2_CO_3_ on device performance.

**Table 1:** Detailed photovoltaic parameters of CsPbI_2_Br PeSCs with different ZnO ETMs measured under 100 mW cm^−2^ AM 1.5G illumination

Substrate	ETM	*V*_OC_ [V]	*J*_SC_ [mA cm^−2^]	FF [%]	PCE (best) [%]	PCE (avg.) [%]
Glass	ZnO	1.13	15.42	72.2	12.68	12.14
	ZnO:Cs_2_CO_3_	1.28	16.34	78.5	16.42	16.10
	ZnO:CsF	1.20	15.92	74.8	14.34	13.71
	ZnO:K_2_CO_3_	1.10	15.85	65.5	11.48	10.86
Plastic	ZnO:Cs_2_CO_3_	1.21	16.11	75.4	14.82	14.35

**Figure 4:**
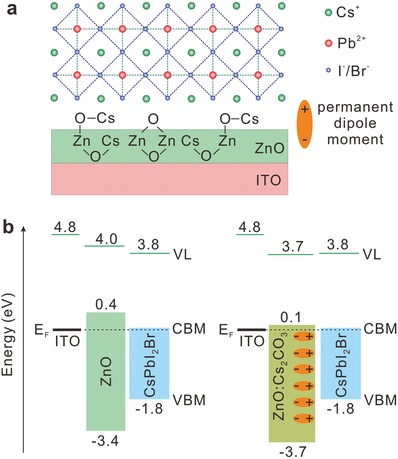
a) Dipole formation at the ZnO:Cs_2_CO_3_/perovskite interface. b) Energy level alignments of CsPbI_2_Br perovskite layers on bare ZnO and ZnO:Cs_2_CO_3_ ETMs, respectively.

Furthermore, the significant PCE enhancement in CsPbI_2_Br PeSCs based on doped ZnO ETMs arises mainly from the substantial boost in *V*_OC_. Particularly, the use of ZnO:Cs_2_CO_3_ results in the largest *V*_OC_ of 1.28 V, which is 0.15 V larger than that of bare ZnO‐based PeSC. Such an increase in *V*_OC_ is primarily attributed to the more favorable energy level alignment at the interface between ZnO:Cs_2_CO_3_ and CsPbI_2_Br. To further elucidate the role of Cs_2_CO_3_ on device performance, a schematic mechanism image is illustrated in **Figure** **4**a. According to the UPS and XPS results (Figure 1b,c), the Cs_2_CO_3_ doping in ZnO leads to the partial substitution of the Zn^2+^ ions with Cs^+^, and thereby an increase in the dangling bond of O^2−^. After the combination of dangling bonds and dissociative Cs^+^ ions, the oriented bonds with a permanent dipole moment pointing to Zn^2+^ are formed at the ZnO surface, showing a decrease in φ_ZnO_ from 4.0 to 3.7 eV. Compared to bare ZnO, the Cs_2_CO_3_ doping contributes to an extra vacuum level (VL) shift from 4.0 to 3.7 eV. Figure 4b depicts the energy level diagrams of the CsPbI_2_Br perovskite layers on bare ZnO and ZnO:Cs_2_CBO_3_ ETMs, respectively, where the energy levels are aligned with a common Fermi level (*E*_F_) by taking into account the VL offsets at the interface. The VL and valence band maximum (VBM) were extracted from the UPS measurements, while the conduction band minimum (CBM) was determined by considering the optical gaps. It is clear that a matched energy level alignment between the CBMs of ZnO:Cs_2_CO_3_ and CsPbI_2_Br is achieved, which is nearly barrier free for the electron extraction. It is thus expected that the charge accumulation and recombination loss at the interfaces can be significantly suppressed, and the built‐in potential across the perovskite film can be enhanced. Consequently, the resulting *V*_OC_ and FF are largely enhanced due to the Cs_2_CO_3_ doping in ZnO ETM. Electrochemical impedance spectroscopy (EIS) and steady‐state photoluminesence (PL) measurements were further conducted to study the charge extraction behaviors at the interfaces, and the corresponding results are plotted in Figure S8 (Supporting Information). It is found that the ZnO:Cs_2_CO_3_ device exhibits the largest semicircle at the low‐frequency region, suggesting a high recombination resistance as well as the effectively suppressed charge recombination.^26^ Besides, the relative low PL intensity for the CsPbI_2_Br on ZnO:Cs_2_CO_3_ reveals that the charge extraction is strongly promoted due to the defect passivation and the matched energy level alignment. As a comparison, other dopants (i.e., K_2_CO_3_ and CsF) cannot contribute the effective formation of an interfacial dipole at the modified ZnO surface and thus have the poor influence on the interfacial energy level tuning.

**Figure 5:**
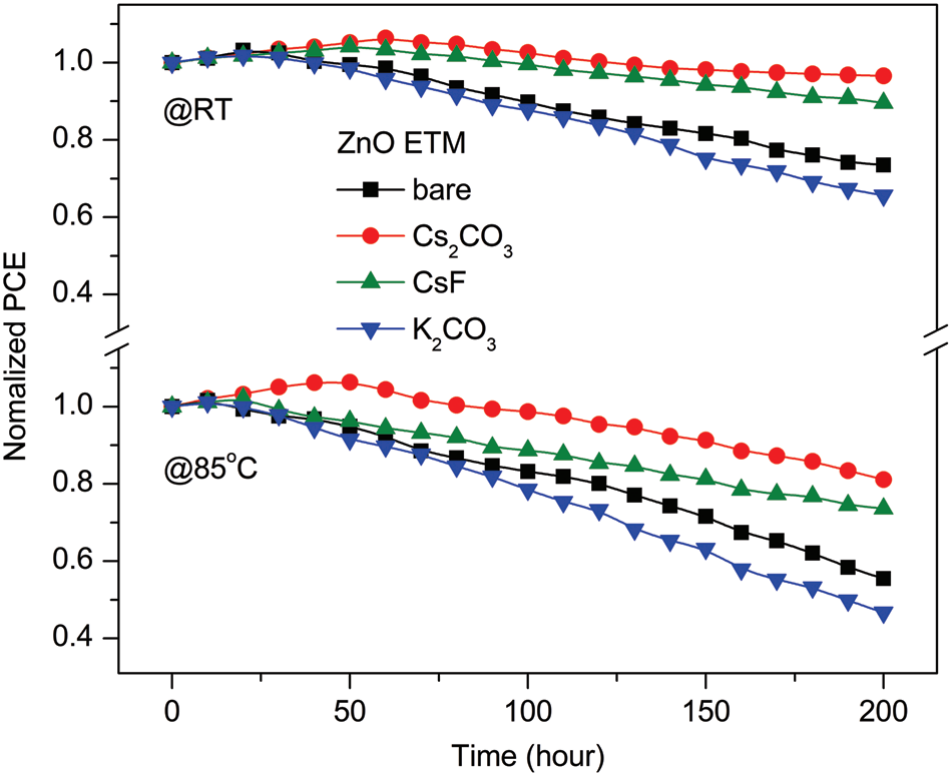
Storing stability of CsPbI_2_Br PeSCs with different ZnO ETMs at room temperature (RT) and 85 °C.

Environmental stability is another key metric for PeSCs, which is highly desirable toward commercial applications. The long‐term storing stability of CsPbI_2_Br PeSCs was monitored by storing two series of un‐encapsulated devices in an N_2_‐filled glovebox in dark at room temperature (RT) and 85 °C, respectively, and periodically recording their photovoltaic performance in air. **Figure** **5** shows the normalized PCEs of CsPbI_2_Br PeSCs with various ZnO ETMs as a function of storage duration. It is noted that the PeSC with a ZnO:Cs_2_CO_3_ ETM exhibits the best storing stability, while the significant PCE drop can be observed for devices with bare ZnO and ZnO:K_2_CO_3_ ETMs. After 200 h of storage at room temperature, CsPbI_2_Br PeSCs based on bare ZnO, ZnO:Cs_2_CO_3_, ZnO:CsF, and ZnO:K_2_CO_3_ retain 73%, 96%, 89%, and 65% of their initial efficiencies, respectively. Moreover, the PeSCs under continuous heating at 85 °C show a degradation trend similar to that stored at room temperature. The use of ZnO:Cs_2_CO_3_ gives rise to the best thermostability for the PeSC stored at 85 °C, which maintains 81% of its initial PCE after 200 h of high‐temperature storage. On the contrary, the bare ZnO‐based device displays a fast decline in PCE, and only 55% of the initial value can be kept under the same storage conditions. It is found in Figure S9 (Supporting Information) that the performance degradation of various PeSCs is mainly related to a severe decrease in *J*_SC_, which can be attributed to the transformation of CsPbI_2_Br crystals from the α‐phase to the δ‐phase.^40^ These results demonstrate that the Cs_2_CO_3_ doping can effectively suppress the phase transformation of CsPbI_2_Br, which might originate from the defect passivation effect of Cs_2_CO_3_ on CsPbI_2_Br crystal surface for improving the long‐term stability.

**Figure 6:**
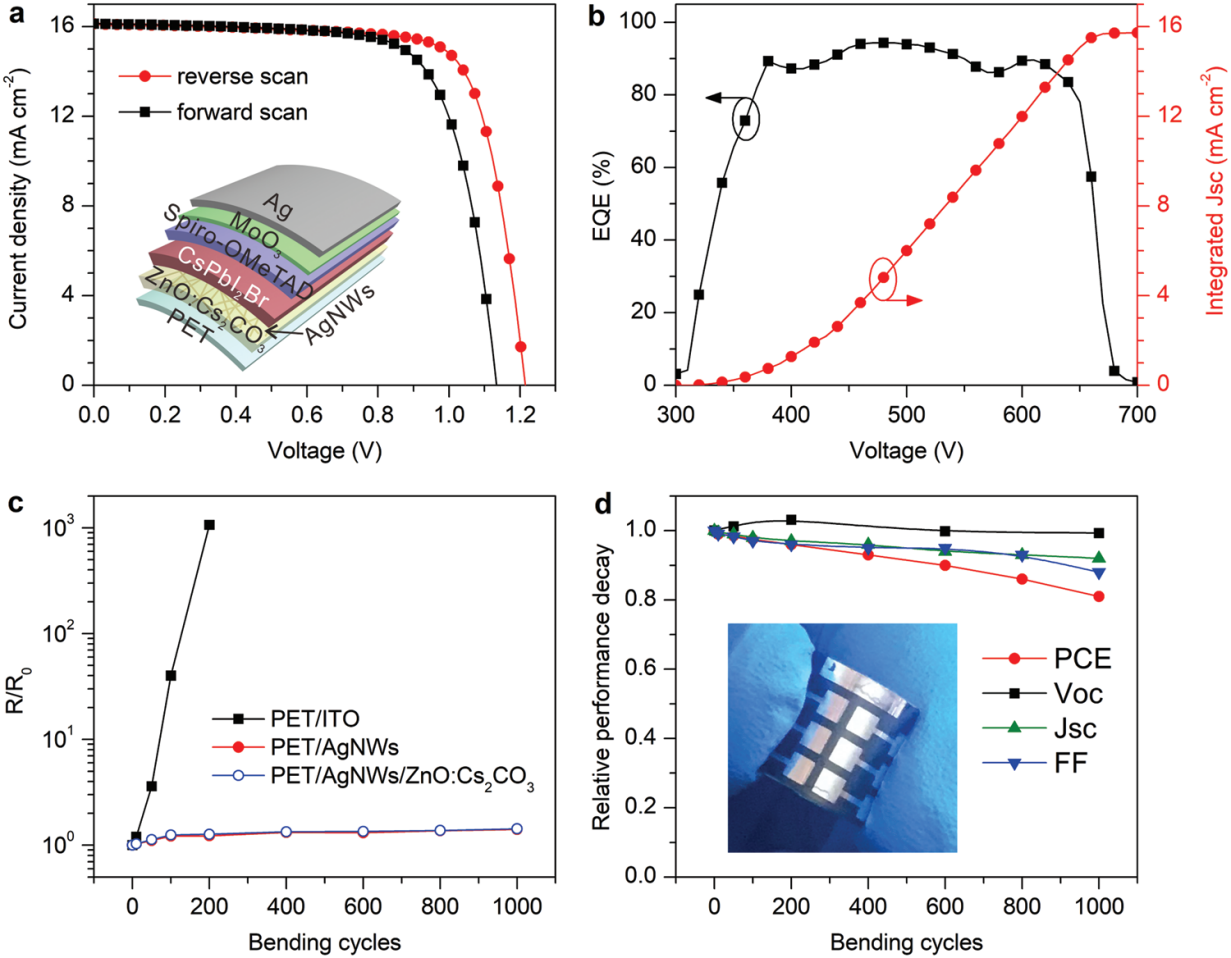
a) *J*–*V* characteristics of flexible PeSC on PET/AgNWs substrate measured in forward and reverse scans. Inset is the schematic of the device architecture. b) EQE spectrum and integrated *J*_SC_ of flexible PeSC. c) *R*/*R*_0_ versus bending cycles for PET/ITO, PET/AgNWs, and PET/AgNWs/ZnO:Cs_2_CO_3_. Bending radius is 8 mm. d) Performance decays versus bending cycles for flexible PeSCs. Inset is a photograph of the bent device.

Given that the CsPbI_2_Br PeSCs can be fabricated with annealing temperatures lower than 150 °C, it is expectable to expand such a device architecture to flexible substrates. We also replaced the rigid ITO–glass substrate with flexible polyethylene terephthalate/Ag nanowires (PET/AgNWs) substrate, which was prepared according to the previous report.^32^ The *J*–*V* characteristics of the representative flexible CsPbI_2_Br PeSC are illustrated in **Figure** **6**a, and the detailed photovoltaic parameters are summarized in Table 1. Under reverse scan, the maximum PCE of 14.82% was achieved with a *V*_OC_ of 1.21 V, *J*_SC_ of 16.11 mA cm^−2^, and FF of 75.4%, while a PCE of 13.21% at the forward scan was obtained. According to the EQE spectrum of the flexible device in Figure 6b, the calculated *J*_SC_ is 15.77 mA cm^−2^, which is in an acceptable mismatch with the experimental result. Furthermore, the mechanical flexibility under bending stress was characterized for various substrates and related devices. Figure 6c plots the increment ratio (*R*/*R*_0_) of sheet resistance as a function of bending cycles at a bending radius of 8 mm. Notably, the *R*/*R*_0_ of PET/ITO substrate rises rapidly to an extremely high value over 1000 Ω sq^−1^ under the repeated bending, while the sheet resistances of PET/AgNWs and PET/AgNWs/ZnO:Cs_2_CO_3_ keep almost constant after 1000 bending cycles. These results indicate that the PET/AgNWs substrate is repeatedly bendable as compared to the commercial PET/ITO substrate, and the coating of ZnO:Cs_2_CO_3_ ETM has negligible influence on mechanical properties. Figure 6d shows the relative performance decay of the complete flexible PeSC as a function of the bending cycles. Encouragingly, flexible CsPbI_2_Br PeSC retains 81% of its initial PCE even after 1000 cycles of repeated bending, indicating that the device is stable against the mechanical stress.

**Figure 7:**
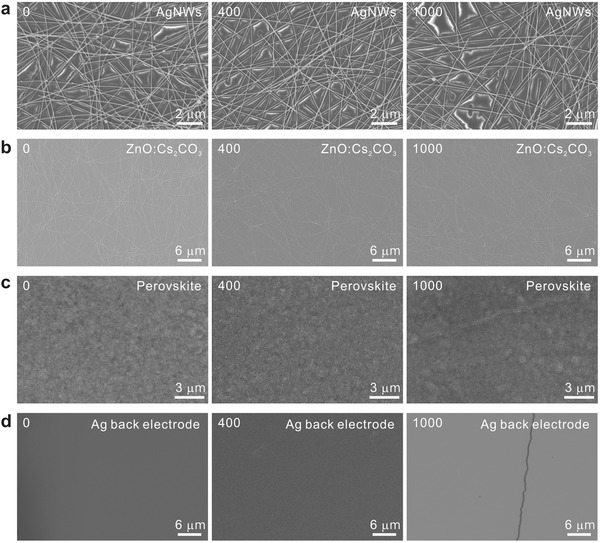
SEM images of a) AgNWs on PET, b) ZnO:Cs_2_CO_3_ on AgNWs, c) CsPbI_2_Br perovskite layer on ZnO:Cs_2_CO_3_, and d) Ag back electrode of the complete PeSC with 0 bending cycle (left column), 400 bending cycles (middle column), and 1000 bending cycles (right column).

To elucidate the key factors affecting the device degradation under bending conditions, the surface morphologies of various films were characterized. **Figure** **7** displays the SEM images of PET/AgNWs, PET/AgNWs/ZnO:Cs_2_CO_3_, CsPbI_2_Br perovskite layers on ZnO:Cs_2_CO_3_, and Ag back electrode of the complete device after different bending cycles. No obvious distinction between pristine and bent samples can be found for PET/AgNWs (Figure 7a) and PET/AgNWs/ZnO:Cs_2_CO_3_ (Figure 7b), revealing the excellent flexibility of the electrodes against mechanical bending. However, cracks will occur on the CsPbI_2_Br perovskite layer (Figure 7c) and Ag back electrode (Figure 7d) after 1000 bending cycles. It turns out that the device degradation after mechanical bending is mainly due to the emerging cracks on the perovskite film under physical deformation. Therefore, further works are required to solve the brittle nature of perovskite films for the realization of highly flexible PeSCs.

## 3 Conclusion

In summary, we have demonstrated a facile route to improving the photovoltaic performance and thermostability of all‐inorganic CsPbI_2_Br PeSCs by tuning the energetics of ZnO ETM via the doping of a classical alkali metal carbonate Cs_2_CO_3_. The formation of an interfacial dipole layer at the doped ZnO/perovskite contact can effectively reduce the energy level offset to a negligible level of 0.1 eV, resulting in the significantly suppressed charge recombination loss and the enhanced built‐in potential across the perovskite layer. The optimized CsPbI_2_Br PeSCs achieve the impressive PCEs of 16.42% and 14.82% on rigid glass and flexible plastic substrates, respectively. Moreover, a high thermostability of CsPbI_2_Br against phase transformation can be simultaneously obtained with a less than 20% efficiency drop for 200 h at 85 °C due to the Cs_2_CO_3_‐assisted defect passivation effect at the doped ZnO/CsPbI_2_Br interface. We anticipate that the sol–gel‐derived ZnO film can be effectively engineered as a competitive alternative to other ETMs in perovskite optoelectronic devices, especially for applications that require low‐temperature processing, such as flexible perovskite photovoltaics.

## 4 Experimental Section

*Chemicals*: Lead iodide (PbI_2_, 99.999%), cesium iodide (CsI, 99.999%), and chlorobenzene (99.9%) were purchased from Alfa Aesar. Lead bromide (PbBr_2_, 99.8%) was purchased from TCI. Zinc acetate dehydrate (99.5%), ethanolamine, and 2‐methoxyethanol (99.8%) were purchased from Sigma Aldrich. Cesium fluoride (≥99.0%), cesium carbonate (≥99.0%), and potassium carbonate (≥99.0%) were purchased from J&K. Dimethyl sulfoxide (DMSO, ≥99.9%) was purchased from Aladdin. Bis(trifluoromethane) sulfonamide lithium salt (99.95%) was purchased from Xi'an p‐OLED.

*Materials' Preparation*: The sol–gel ZnO precursor solution was prepared by following the previous report.^41^ The dopants including CsF, Cs_2_CO_3_, and K_2_CO_3_ were then added into the sol–gel ZnO precursor solution; the concentrations of various dopants were precisely tuned from 2 mol% to 6 mol%. The precursor solution of all‐inorganic mixed‐halide CsPbI_2_Br perovskites was prepared by dissolving 0.694 g of PbI_2_, 0.4840 g of PbBr_2_, and 0.704 g of CsI in 2 mL of DMSO with stirring at 65 °C until the total dispersion. The hole transport material (HTM) solution was prepared by dissolving 72.3 mg of Spiro‐OMeTAD, 29 µL of 4‐*tert*‐butylpyridine (TBP, 96%), 18 µL of lithium bis(trifluoromethanesulfonyl) imide (LiTFSI, 99.95%)/acetonitrile (520 mg mL^−1^) in 1 mL of chlorobenzene (99.9%).

*Device Fabrication*: For rigid PeSCs, the patterned ITO‐coated glass with a sheet resistance of 14 Ω sq^−1^ was used as the rigid substrate, which was sequentially cleaned by detergent, acetone, ethyl alcohol, and isopropanol under ultrasonication for about 15 min in each step, respectively. After drying with N_2_ flow, the ITO–glass substrates were heated in an oven at 110 °C. Prior to the device fabrication, the ITO–glass substrates were treated by ultraviolet ozone for 15 min. The sol–gel ZnO precursor solutions with different dopants were spin‐coated on the ITO–glass substrates at 3000 rpm for 40 s, and annealed at 150 °C for 10 min to obtain the ETMs with a thickness of ≈40 nm. Then, the samples were transferred into a nitrogen‐filled glove box, and the filtered CsPbI_2_Br precursor solution (50 µL) was dropped on different ZnO ETMs via a two‐step program at 500 rpm for 5 s and 2500 rpm for 30 s, respectively. The perovskite films were left at room temperature for 7 min, and then placed onto a hotplate at 150 °C for 8 min to remove the DMSO solvent and promote the further crystallization of perovskites. A CsPbI_2_Br film with a thickness of ≈450 nm was obtained on the ETM after this fabrication process. When the samples were cooled down to room temperature, a Spiro‐OMeTAD layer of ≈200 nm was deposited on the perovskite films by spin‐coating the solution at 4000 rpm for 30 s. Finally, the samples were transferred into the interconnected high‐vacuum evaporation system (base pressure ≈ 2 × 10^−6^ Torr), in which an 8 nm thick MoO_3_ and 110 nm thick Ag were thermally deposited onto the Spiro‐OMeTAD HTM through a shadow mask. The effective area of PeSCs was determined to be 0.0725 cm^2^ by the aperture mask. For flexible devices, diluted AgNW dispersion ink (2 mg mL^−1^) was spin‐coated on UV–ozone‐treated PET substrate twice at 1500 rpm for 30 s, and then dried at 100 °C for 5 min. Then, the sol–gel ZnO precursor solution was spin‐coated twice on the AgNW‐based electrode at 3000 rpm for 40 s, and annealed at 150 °C for 10 min to form a smooth film. Then, CsPbI_2_Br, Spiro‐OMeTAD, MoO_3_, and Ag layers were successively deposited on the ZnO ETMs in the same way to the rigid devices.

*Films and Device Characterization*: Chemical states, valence states, and VL of various ZnO ETMs were characterized by XPS and UPS (Kratos AXIS Ultra DLD).^42^ The UPS measurements were performed with a He I (*hν* = 21.22 eV) gas discharge lamp for excitation and a total instrumental energy resolution of 100 meV, and samples were negatively biased for the observation of SECOs. XPS spectra were obtained with a monochromatic Al Kα source (1486.6 eV) with a resolution of 0.5 eV. All the UPS and XPS spectra were referred to Fermi level (*E*_F_) as zero binding energy. Absorption and transmission measurements were conducted using a UV–vis–near‐IR spectrometer (Perkin Elmer Lambda 950). Film morphologies of various ZnO ETMs and perovskite films were characterized by a scanning electron microscope (Zeiss G500). Crystallographic properties of perovskite films were recorded by XRD with monochromatic Cu Kα radiation. Steady‐state PL and transient PL lifetime of perovskite films were collected by a FluoroMax‐4 fluorescence spectrometer (Horiba Jobin Yvon) and a Horib‐FM‐2015 spectrometer, respectively. Photovoltaic characteristics of PeSCs were performed by a programmable Keithley 2612 source measurement unit under the illumination of 1 sun, air mass (AM) 1.5G spectrum from a 150 W Newport 91 160 solar simulator, which was calibrated to be 100 mW cm^−2^ by standard Si reference cell with a known spectral response. The EQE spectra were measured with a photo‐modulation spectroscopic setup (Newport monochromator).

## Conflict of Interest

The authors declare no conflict of interest.

## Supporting information

Supplementary
