# Supplementary material for: Interfacial Energy Level Tuning for Efficient and Thermostable CsPbI2Br Perovskite Solar Cells
Source: Adv Sci (Weinh). 2019 Sep 30;7(1):1901952. doi: 10.1002/advs.201901952 (PMC6947708; doi:10.1002/advs.201901952)
Supplement: Supplementary file 1 — Supplementary [file ADVS-7-1901952-s001.pdf]

Copyright WILEY-VCH Verlag GmbH & Co. KGaA, 69469 Weinheim, Germany, 2019.

ADVANCED SCIENCE
Open Access

Supporting Information

for *Adv. Sci.*, DOI: 10.1002/advs.201901952

Interfacial Energy Level Tuning for Efficient and Thermostable $CsPbI_2Br$ Perovskite Solar Cells

En-Chi Shen, Jing-De Chen, Yu Tian, Yu-Xin Luo, Yang Shen, Qi Sun, Teng-Yu Jin, Guo-Zheng Shi, Yan-Qing Li,* and Jian-Xin Tang**

Supporting Information

Interfacial Energy Level Tuning for Efficient and Thermostable $CsPbI_2Br$ Perovskite Solar Cells

En-Chi Shen, Jing-De Chen,* Yu Tian, Yu-Xin Luo, Yang Shen, Qi Sun, Teng-Yu Jin, Guo-Zheng Shi, Yan-Qing Li,* Jian-Xin Tang*

E. C. Shen, Dr. J. D. Chen, Y. Tian, Y. X. Luo, Y. Shen, Q. Sun, T. Y. Jin, Dr. G. Z. Shi, Prof. Y. Q. Li, Prof. J. X. Tang
Jiangsu Key Laboratory for Carbon-Based Functional Materials & Devices
Institute of Functional Nano & Soft Materials (FUNSOM)
Soochow University
199 Ren'ai Road, 215123, Suzhou, Jiangsu, PR China
E-mail: jdchen@suda.edu.cn (J.D. Chen); yqli@suda.edu.cn (Y.Q. Li); jxtang@suda.edu.cn (J.X. Tang)

Prof. Y. Q. Li
School of Physics and Electronics Science
Ministry of Education Nanophotonics & Advanced Instrument Engineering Research Center
East China Normal University
Shanghai, 200062, China

Prof. J. X. Tang
Institute of Organic Optoelectronics (IOO), JITRI
Wujiang, 215215, Suzhou, China

Keywords: all-inorganic perovskite solar cells; energy level alignment; thermal stability; flexible perovskite solar cells

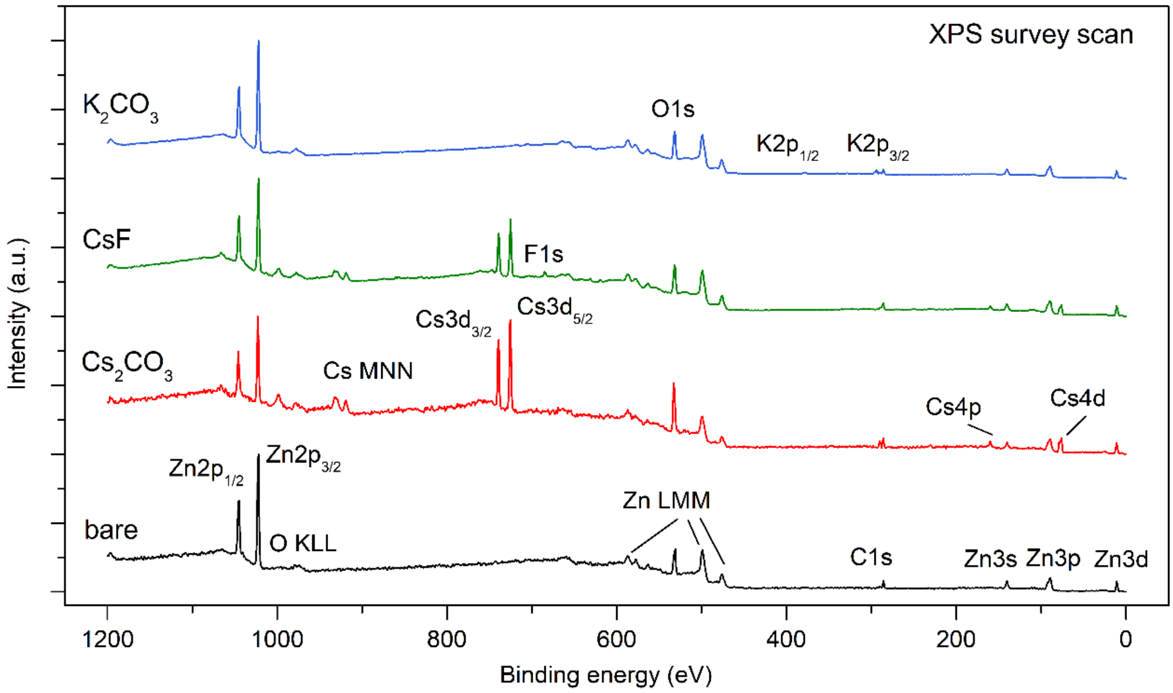

Figure S1. XPS survey scans of ZnO ETMs modified with different dopants: a) bare, b) Cs_2CO_3, c) CsF, and d) K_2CO_3.

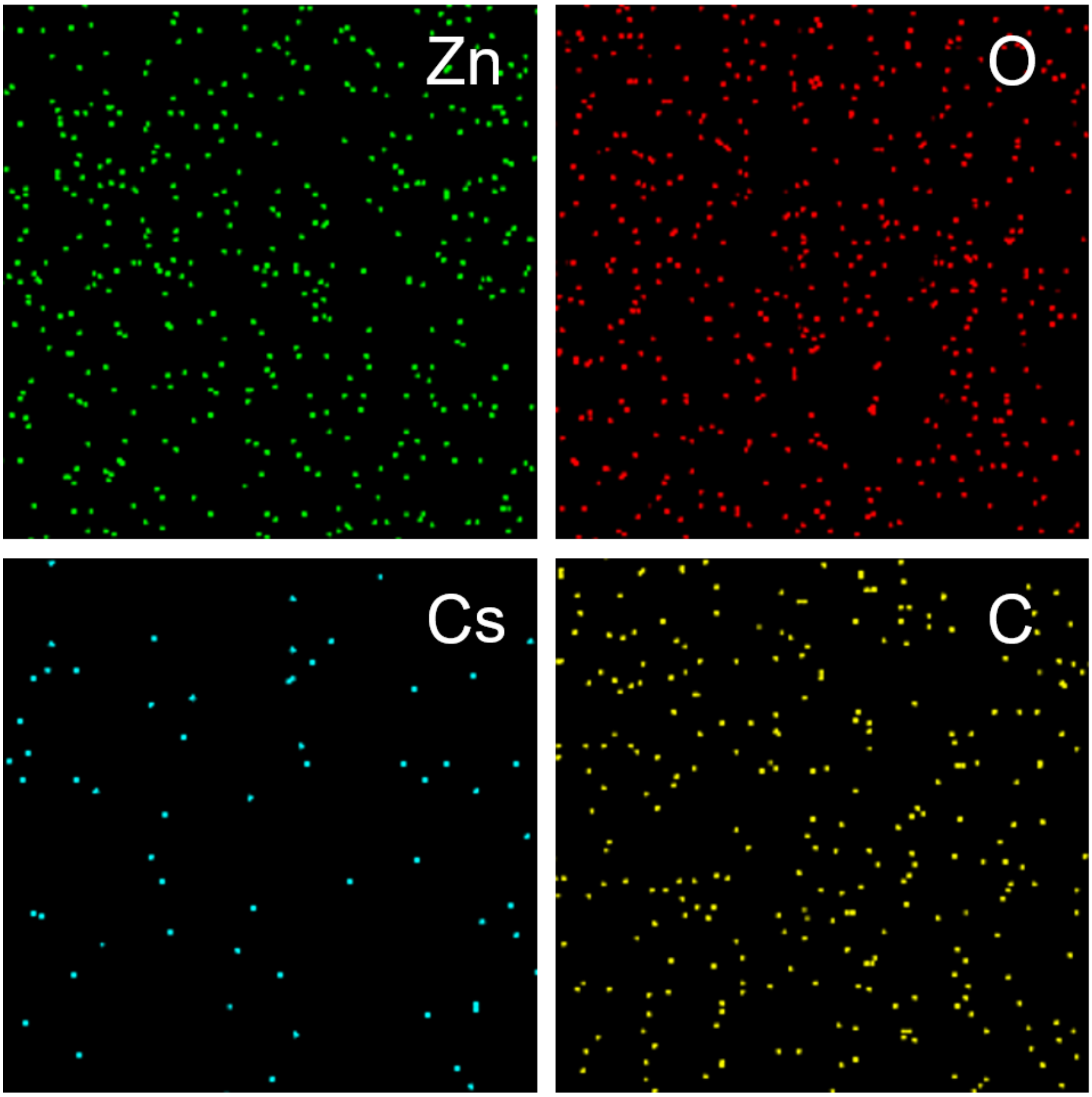

Figure S2. EDX images of ZnO:Cs_2CO_3 ETM.

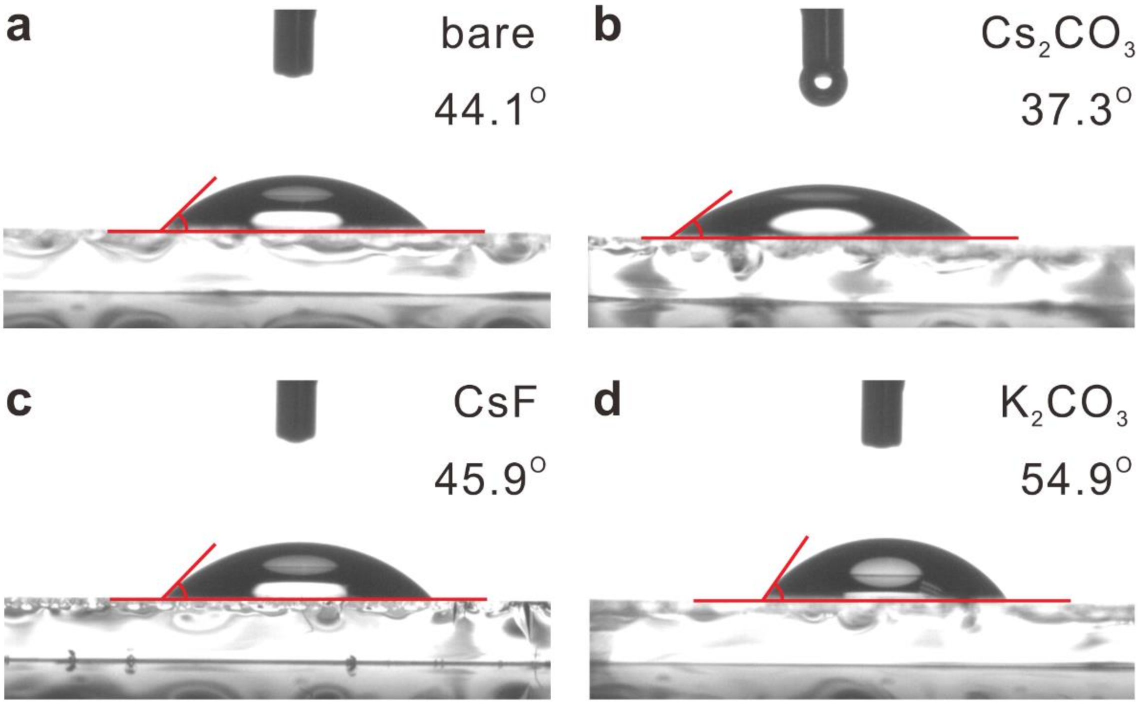

Figure S3. Images of contact angle measurements for droplets on a) bare ZnO, b) ZnO:Cs_2CO_3, c) ZnO:CsF, and d) ZnO:K_2CO_3.

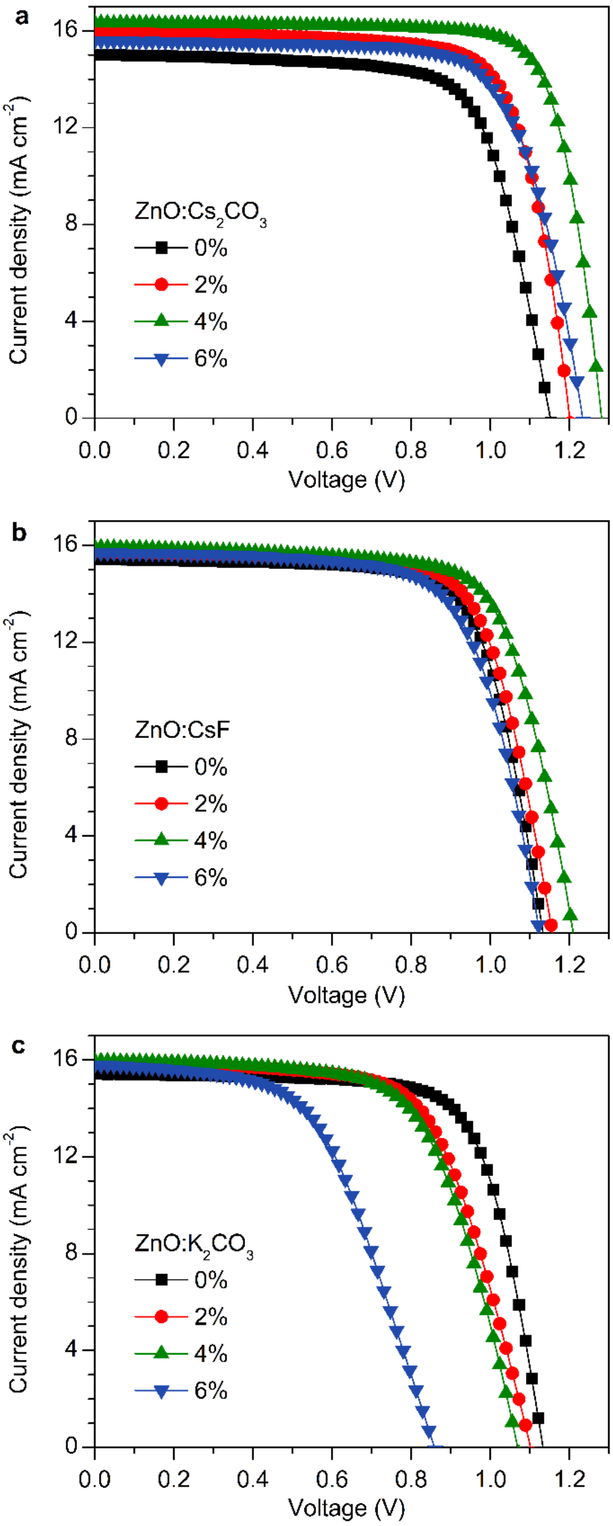

Figure S4. J-V characteristics of $CsPbI_2Br$ PeSCs based on a) ZnO:Cs_2CO_3, b) ZnO:CsF, and c) ZnO:K_2CO_3 ETMs scanned in reverse direction under 100 mW cm^{-2} AM 1.5G illumination.

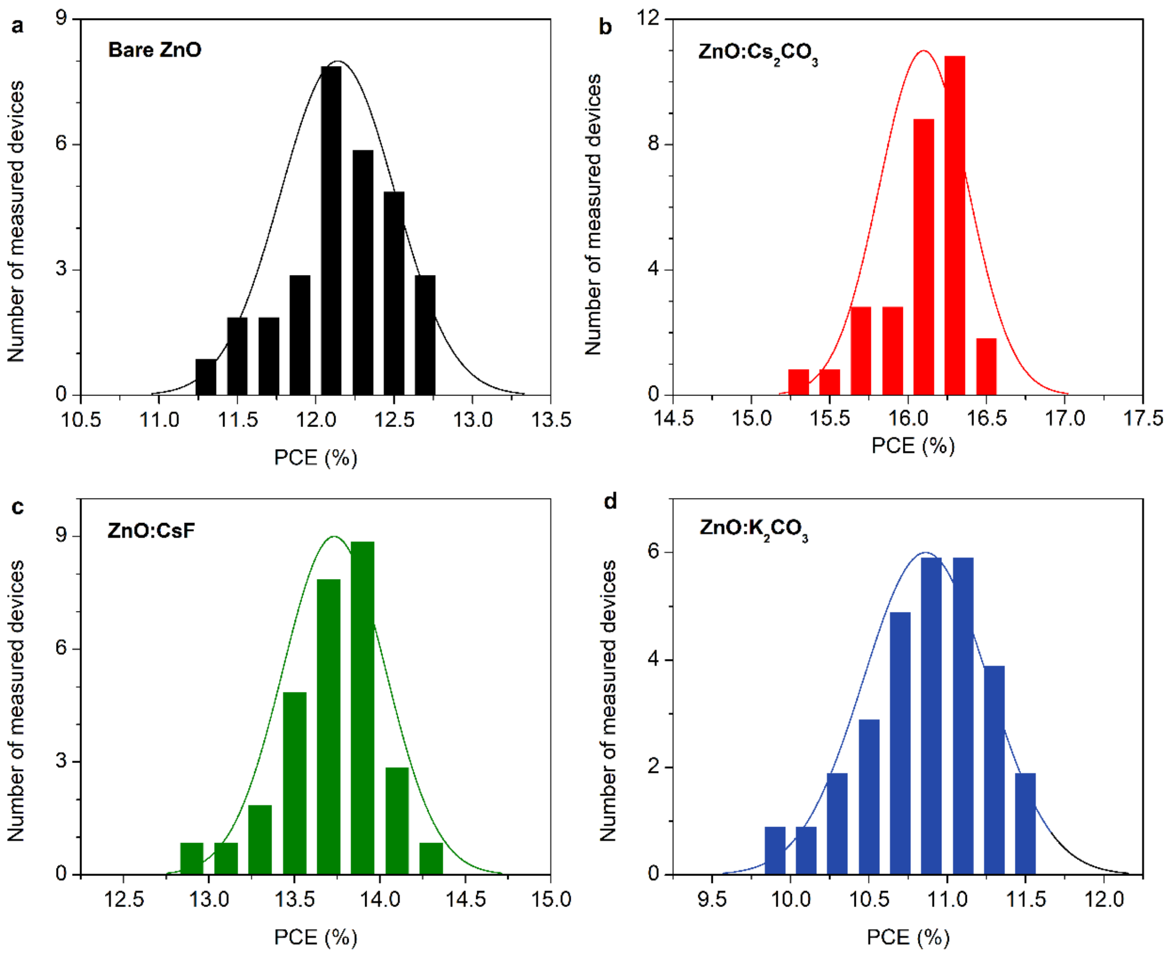

Figure S5. Histograms of PCE values of $CsPbI_2Br$ PeSCs with different ZnO ETMs.

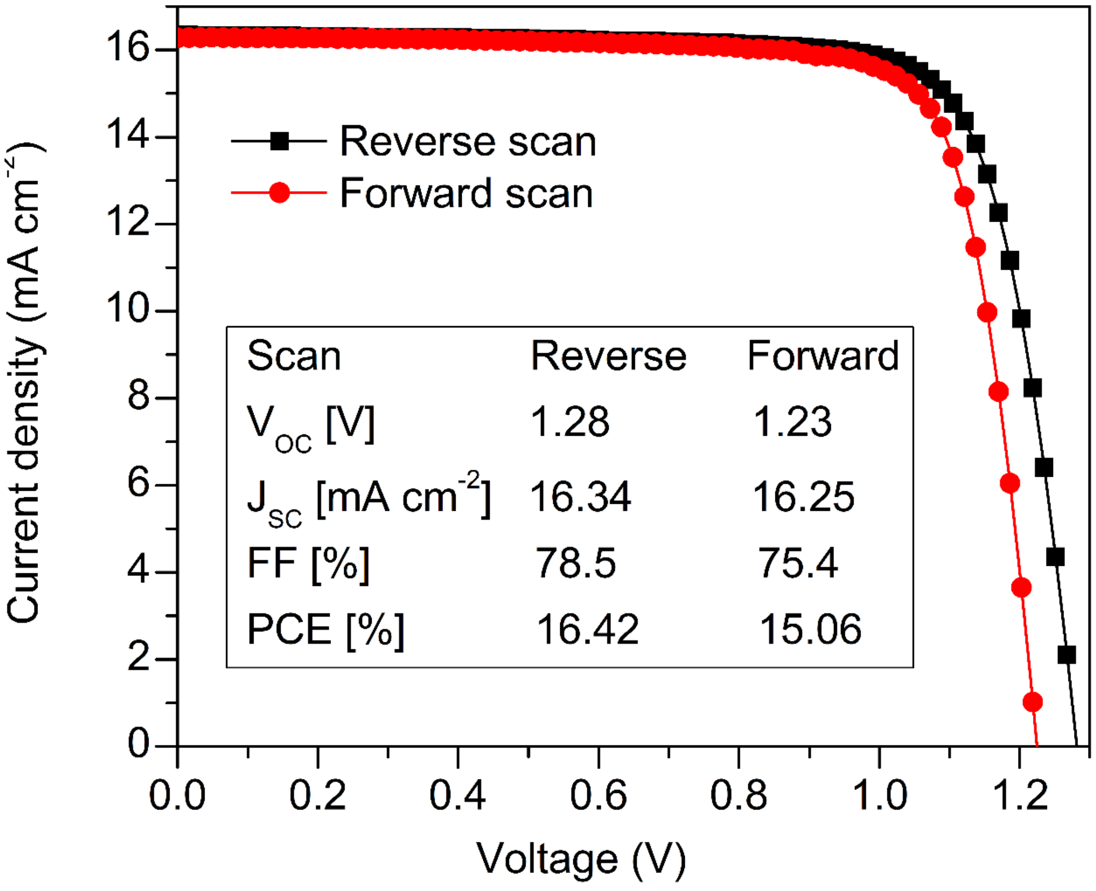

Figure S6. J-V characteristics of $CsPbI_2Br$ PeSC on $ZnO:Cs_2CO_3$ (4 mol%) ETM in reverse and forward scans.

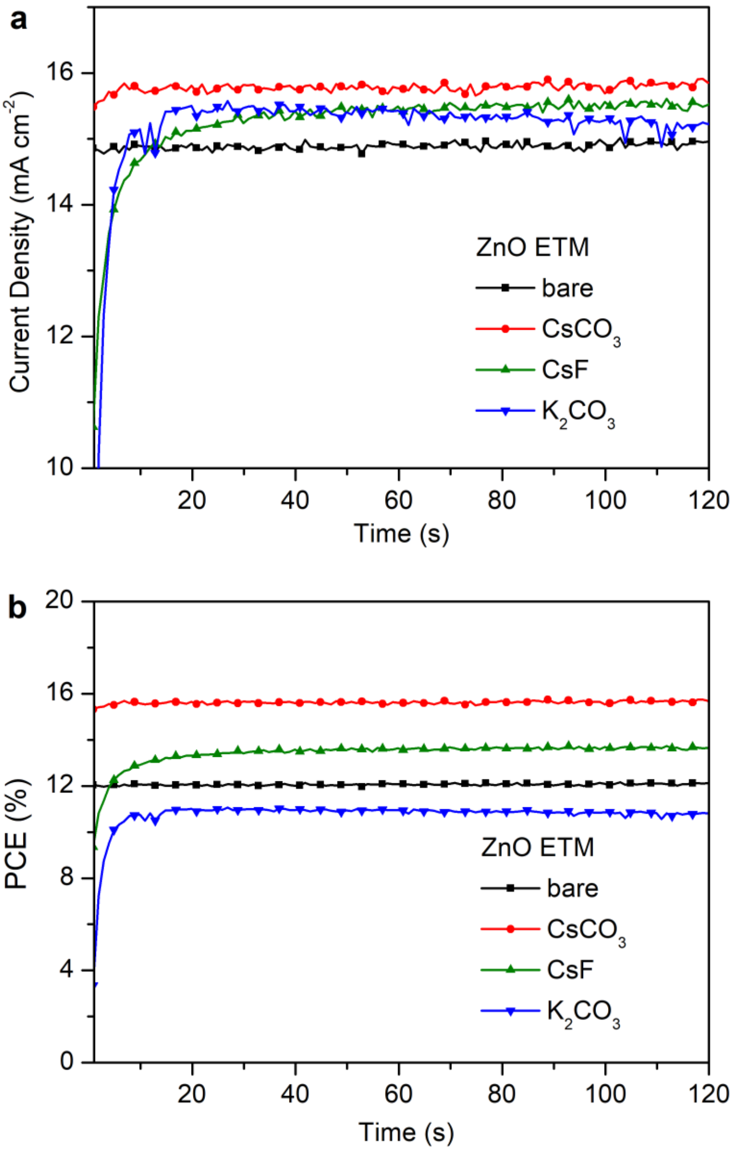

Figure S7. Stabilized power output (SPO) curves of various PeSCs at the maximum power.

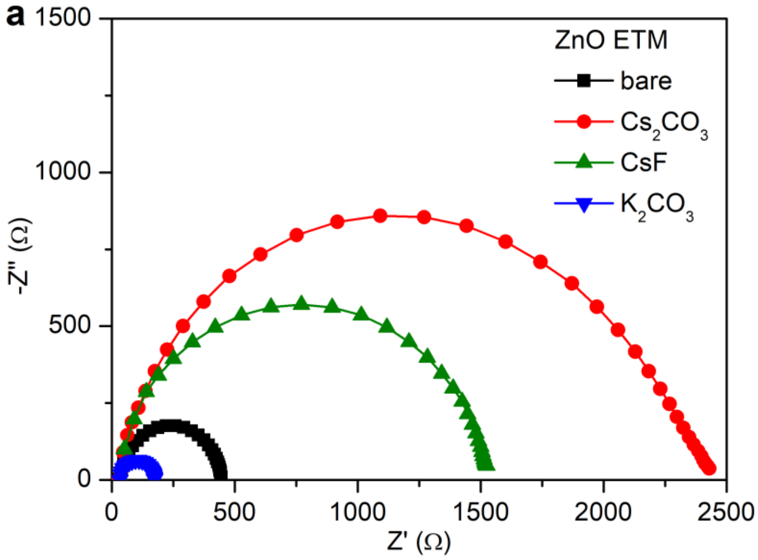

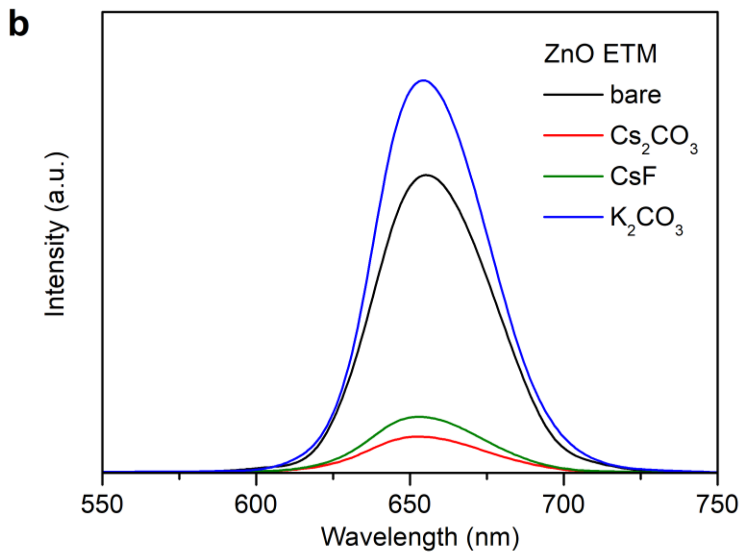

Figure S8. a) Nyquist plots of PeSCs with various ETMs. b) Steady-state PL spectra of $CsPbI_2Br$ deposited on various ETMs.

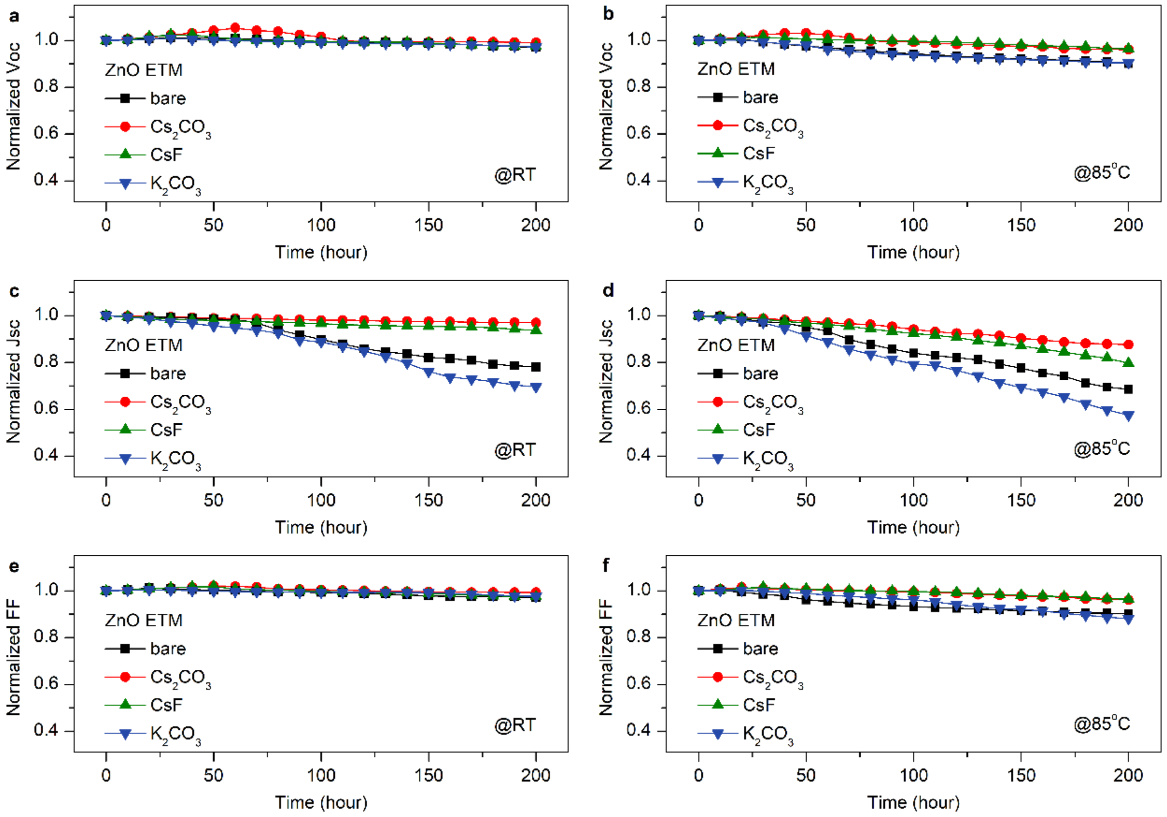

Figure S9. Storing stability of $CsPbI_2Br$ PeSCs with various doped ZnO ETMs at room temperature (RT) (left column) and 85 °C (right column).
